# Feedback activation of STAT3 mediates trastuzumab resistance via upregulation of MUC1 and MUC4 expression

**DOI:** 10.18632/oncotarget.2135

**Published:** 2014-06-26

**Authors:** Guangchao Li, Likun Zhao, Wei Li, Kexing Fan, Weizhu Qian, Sheng Hou, Hao Wang, Jianxin Dai, Huafeng Wei, Yajun Guo

**Affiliations:** ^1^ School of Bioscience and Bioengneering, South China University of Technology, Guangzhou, China; ^2^ International Joint Cancer Institute, Second Military Medical University, Shanghai, China; ^3^ State Key Laboratory of Antibody Medicine & Targeting Therapy and Shanghai Key Laboratory of Cell Engineering & Antibody, Shanghai, China; ^4^ School of Pharmacy, Liaocheng University, Liaocheng, Shandong Provence, China

**Keywords:** HER2, trastuzumab resistance, STAT3, MUC1/4, feedback loop

## Abstract

Although HER2-targeting antibody trastuzumab confers a substantial benefit for patients with HER2-overexpressing breast and gastric cancer, overcoming trastuzumab resistance remains a large unmet need. In this study, we revealed a STAT3-centered positive feedback loop that mediates the resistance of trastuzumab. Mechanistically, chronic exposure of trastuzumab causes the upregulation of fibronection (FN), EGF and IL-6 in parental trastuzumab-sensitive breast and gastric cells and convergently leads to STAT3 hyperactivation. Activated STAT3 enhances the expression of FN, EGF and IL-6, thus constituting a positive feedback loop which amplifies and maintains the STAT3 signal; furthermore, hyperactivated STAT3 signal promotes the expression of MUC1 and MUC4, consequently mediating trastuzumab resistance via maintenance of persistent HER2 activation and masking of trastuzumab binding to HER2 respectively. Genetic or pharmacological inhibition of STAT3 disrupted STAT3-dependent positive feedback loop and recovered the trastuzumab sensitivity partially due to increased apoptosis induction. Combined trastuzumab with STAT3 inhibition synergistically suppressed the growth of the trastuzumab-resistant tumor xenografts *in vivo*. Taken together, our results suggest that feedback activation of STAT3 constitutes a key node mediating trastuzumab resistance. Combinatorial targeting on both HER2 and STAT3 may enhance the efficacy of trastuzumab or other HER2-targeting agents in HER2-positive breast and gastric cancer.

## INTRODUCTION

Human epidermal growth factor receptor 2 (HER2) is a receptor tyrosine kinase (RTK) that regulates cell growth and differentiation signaling pathways and is greatly overexpressed in approximately 20% to 25% of breast [1, 2] and 30% of gastric cancers [3], leading to an aggressive tumor phenotype and dismal prognosis. Trastuzumab, a humanized antibody directed against the extracellular domain of the HER2 receptor, shows considerable clinical efficacy and improves survival in patients with HER2-positive breast cancer [4-6]. It has been approved by the U.S. Food and Drug Administration (FDA) for use in early-stage and metastatic HER2-positive breast cancers as well as in metastatic, unresectable HER2-positive gastric and gastroesophageal junction cancers [7].

Despite the survival benefits gained from trastuzumab therapy, many patients with metastatic HER2-positive breast cancer do not respond to initial trastuzumab treatment (*de novo* resistance) and the majority of trastuzumab-responsive patients develop resistance within 1 year of treatment initiation [8, 9]. A number of resistance mechanisms have been proposed: (i) aberrant activation of the PI3K/AKT pathway due to phosphatase and tensin homolog (PTEN) deficiency or *PIK3CA* gene activating mutations [10, 11], (ii) alternative activation of other RTK signals [12-15], (iii) the accumulation of truncated HER2 receptors (p95HER2) that lacks the trastuzumab-binding domain [16], (iv) downregulation of p27(kip1) level [17], and (v) cyclin E amplification/overexpression[18]. Although these findings provide considerable insights into the trastuzumab resistance, additional mechanisms remain to be identified, and further studies are also needed to explore whether similar resistance mechanisms are operative in breast and gastric cancer.

We have previously established two trastuzumab-resistant cell lines (BT474R and NCI-N87R) respectively derived from HER2-overexpressing breast and gastric cancer cell lines (BT474 and NCI-N87) *in vitro* by continuously culturing parental cells with increasing dose of trastuzumab for a long period of time and found that these two resistant cells displayed a markedly enhanced phosphorylation of signal transducer and activator of transcription-3 (STAT3) compared to parental cells (unpublished data). STAT3 is a latent cytoplasmic transcription factor that delivers signals from the cell surface to the nucleus in response to extracellular signals, such as cytokines or growth factors [19]. STAT3 is constitutively activated in many types of human cancers and plays crucial roles in regulating tumor cell proliferation, survival, invasion, angiogenesis, and immune evasion [20, 21]. Accumulating evidence has demonstrated that aberrant expression and activity of STAT3 are implicated in both cancer stem cell (CSC) expansion and associated drug resistance in several cancer types, including breast and gastric cancer [22-25], suggesting that STAT3 may contribute to trastuzumab resistance in HER2-positive solid cancer.

In this study, we show that STAT3 phosphorylation is significantly increased in *de novo* and acquired trastuzumab-resistant breast and gastric cancer cells. The increased STAT3 signaling is mediated by elevated expression of fibronection (FN), EGF, and IL-6 in an autocrine manner, which convergently leads to trastuzumab resistance via upregulating the expression of MUC1 and MUC4, two downstream targets of STAT3 capable of inducing trastuzumab resistance via maintaining HER2 activation and masking of trastuzumab binding to HER2 respectively. Notably, abrogation of STAT3 activation by knocking down STAT3 expression or STAT3-specific small-molecule inhibitor recovered the trastuzumab sensitivity of resistant cells *in vitro* and *in vivo*. The data suggest that STAT3 inhibitor in combination with trastuzumab may overcome the trastuzumab resistance in patients with HER2-positive solid tumors exhibiting hyperactivated STAT3 signal.

## RESULTS

### STAT3 hyperactivation in acquired trastuzumab-resistant cells

**Figure 1:**
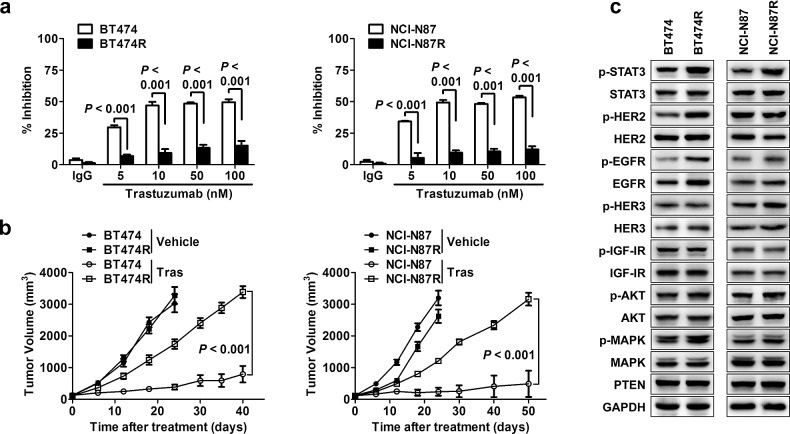
STAT3 hyperactivation in acquired trastuzumab-resistant cells (A) Trastuzumab-sensitive BT474 and NCI-N87 were made resistant by chronic exposure to increasing concentrations of trastuzumab. MTS assay evaluating cell proliferation of the indicated parental cell lines and their corresponding acquired resistant sublines upon treatment with increasing concentrations of trastuzumab (Tras) for 4 d. (B) Tumor growth curves of xenografts derived from either trastuzumab-sensitive or -resistant sublines upon treatment of vehicle or trastuzumab weekly. (C) Immunoblots evaluating major cell signaling changes in the indicated trastuzumab-sensitive and -resistant cells. p indicates phosphorylation. GAPDH blot served as loading controls. Data are expressed as mean ± SD of two independent experiments performed in triplicate samples, and picture is representative of three independent experiments.

We firstly obtained trastuzumab-resistant HER2-amplified BT474R breast cancer cell line and NCI-N87R gastric cancer cell line by culturing parental cells with increasing doses of trastuzumab for over 15 months *in vitro*. Compared with parental cells, BT474R and NCI-N87R exhibited significantly resistance to trastuzumab treatment *in vitro* (Fig. 1A). Similarly, trastuzumab treatment had little effect on *in vivo* growth of subcutaneously established xenografts from BT474R and NCI-N87R cells although evident suppression was seen for the xenografts from parental BT474 and NCI-N87 cells (Fig. 1B). Correspondingly, trastuzumab treatment markedly inhibited the AKT phosphorylation in xenografts from parental BT474 and NCI-N87 cells but not from their corresponding resistant cells as evidenced by immunohistological staining of phosphorylated AKT in excised tumor xenografts (Supplementary Fig. 1).

To probe the molecular alterations underlying trastuzumab resistance, we screened the status of alternative RTKs and their downstream signaling pathways previously implicated in trastuzumab resistance.[12-15] As shown in Fig. 1C, a significant increase in STAT3 phosphorylation (at Tyr705) was noted in both resistant cancer cells compared to their parental cells, which was also evident in tumor xenografts presenting an increased staining of phosphorylated STAT3 (Supplementary Fig. 1). The resistant cells also exhibited an increased EGFR phosphorylation (at Tyr1068), indicating that EGFR signaling may be involved in acquired resistance mechanisms in our model. No changes in PTEN protein and AKT phosphorylation were observed, suggesting that the acquired resistance of BT474R and NCI-N87R cells to trastuzumab was not due to PI3K/AKT pathway enhancement. In addition, the levels of HER2, HER3 and IGF1R proteins and their phosphorylation were unchanged in the resistant cells (Fig. 1C). Collectively, the data suggest that hyperactivation of STAT3 pathway may be a key signaling alteration contributing to acquired trastuzumab resistance in our model.

### STAT3 is hyperactivated in *de novo* trastuzumab-resistant cells

**Figure 2:**
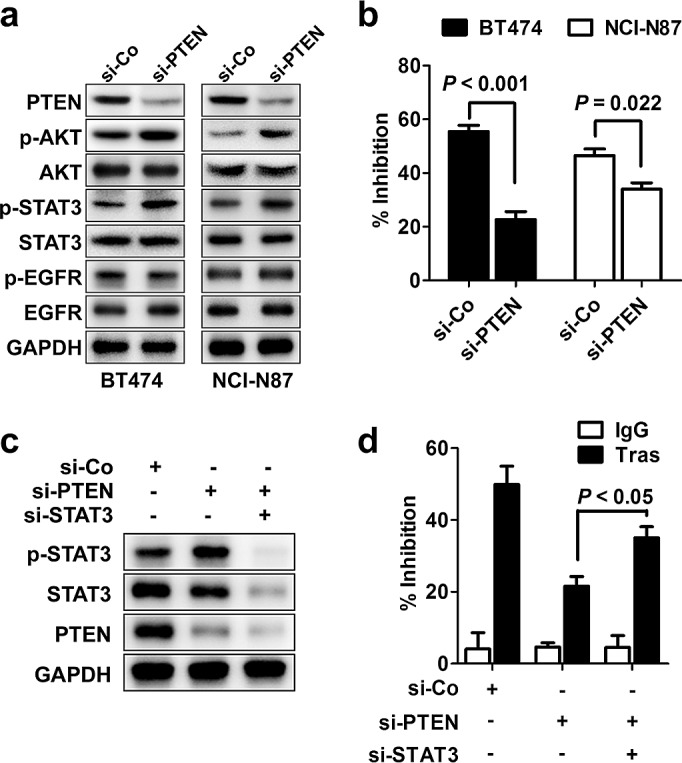
STAT3 hyperactivation in *de novo* trastuzumab-resistant cells (A) Immunoblots assessing the effects of PTEN knockdown on phosphorylation of STAT3 (p-STAT3) and other signals. (B) MTS assay comparing proliferation of cells with transfection of control (si-Co) or PTEN-specific siRNA (si-PTEN) under trastuzumab treatment (Tras, 50 nM, 4 d). (C) Immunoblots evaluating the efficiency of STAT3 knockdown in parental BT474 cells with PTEN depletion. (D) MTS assay assessing trastuzumab sensitivity of PTEN-depleted cells with or without STAT3 knockdown. Data are expressed as mean ± SD of three independent experiments performed in triplicate samples, and picture is representative of two independent experiments.

PTEN deficiency confers *de novo* trastuzumab resistance [11]. We applied PTEN-targeting siRNA (si-PTEN) to successfully knockdown PTEN expression as indicated by decreased PTEN expression and increased AKT phosphorylation (Fig. 2A). As expected, knocking down PTEN expression induced considerable resistance to trastuzumab treatment compared to control cells (Fig. 2B). Notably, although PTEN silencing had no obvious effect on EGFR activation, it led to a prominent increase in STAT3 phosphorylation, indicating that PTEN loss also induced STAT3 hyperactivation (Fig. 2A). Furthermore, silencing STAT3 in PTEN-deficient BT474 parental cells significantly rescued the sensitivity of these cells to trastuzumab treatment (Fig. 2C and 2D). Therefore, STAT3 hyperactivation due to PTEN deficiency contributed to the *de novo* trastuzumab resistance, and such resistance could be overcome by STAT3 inhibition in PTEN-deficient cells.

### STAT3 regulates the sensitivity to trastuzumab treatment

**Figure 3:**
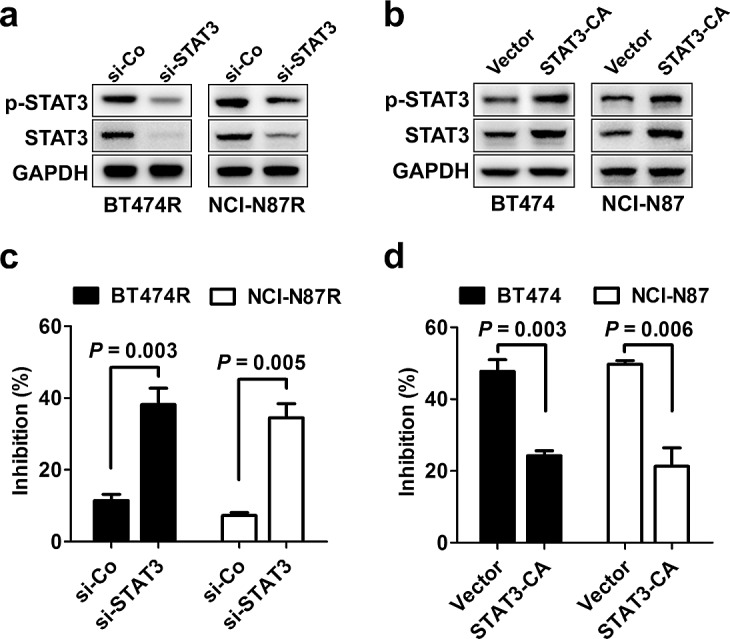
STAT3 activity regulates sensitivity to trastuzumab treatment (A) Immunoblots evaluating STAT3 expression and activation in BT474R and NCI-N87R cells after transfection with control (si-Co) or STAT3-specific siRNA (si-STAT3). (B) Parental BT474 and NCI-N87 cells were transiently transfected with control or STAT3-CA plasmid. After 24 h, STAT3 expression and activation was detected by immunoblotting. (C) MTS assay comparing proliferation of trastuzumab-resistant BT474R and NCI-N87R cells with or without STAT3 knockdown (Tras, 50 nM, 4 d). (D) MTS assay comparing proliferation of trastuzumab-sensitive parental BT474 and NCI-N87 cells with or without constitutive STAT3 activation. Data are expressed as mean ± SD of three independent experiments performed in triplicate samples, and picture is representative of two independent experiments.

To further elucidate the role of STAT3 pathway in trastuzumab resistance, STAT3 was silenced in the two resistant cell lines BT474R and NCI-N87R (Fig. 3A), and the constitutively active form of STAT3 (STAT3-CA) was ectopically expressed in two parental cell lines BT474 and NCI-N87 (Fig. 3B). As shown in Fig. 3C, transfection with STAT3-targeting siRNA, but not control siRNA, decreased the levels of STAT3 expression and phosphorylation in BT474R and NCI-N87R cells, leading to restoration of their sensitivity to trastuzumab treatment *in vitro*. In contrast, parental BT474 and NCI-N87 cells expressing STAT3-CA displayed an evident increase in STAT3 expression and its phosphorylation compared to cells expressing control vector, which made them highly resistant to trastuzumab-mediated growth inhibition (Fig. 3D). The data indicate that STAT3 hyperactivation is sufficient to confer trastuzumab resistance.

### Heterogeneous mediators lead to STAT3 heperactivation contributing to trastuzumab resistance

**Figure 4:**
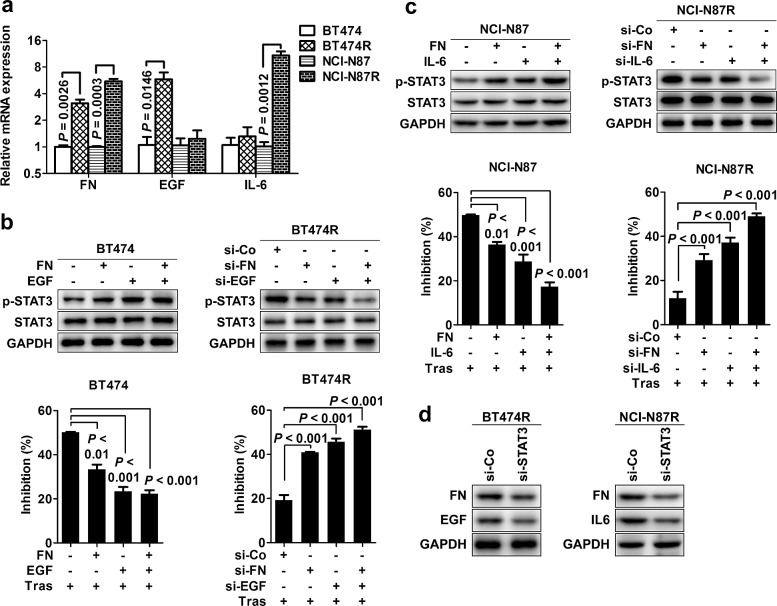
Heterogeneous mediators cause STAT3 heperactivation contributing to trastuzumab resistance (A) Real-time PCR examining the relative mRNA levels of FN, EGF and IL-6 in parental and trastuzumab-resistant cell lines. (B) Left, the effects of either FN or EGF alone or combined stimulations on STAT3 phosphorylation (upper) and cell proliferation upon transtuzumab treatment (bottom) in parental BT474 cells. Right, the effects of either FN or EGF alone or combined knockdown on STAT3 phosphorylation (upper) and cell proliferation upon transtuzumab treatment (bottom) in BT474R cells. (C) Left, the effects of either FN or IL-6 alone or combined stimulations on STAT3 phosphorylation (upper) and cell proliferation upon transtuzumab treatment (bottom) in parental NCI-N87 cells. Right, the effects of either FN or IL-6 alone or combined knockdown on STAT3 phosphorylation (upper) and cell proliferation upon transtuzumab treatment (bottom) in NCI-N87R cells. (D) Immunoblots evaluating FN, EGF or IL-6 expression in BT474R and NCI-N87R cells after STAT3 knockdown. Data are expressed as mean ± SD of three independent experiments performed in triplicate samples, and picture is representative of three independent experiments.

Our results demonstrate that the STAT3 hyperactivation causes trastuzumab resistance; however, upstream mediators leading to STAT3 hyperactivation remain unclear. We screened the expression of 91 known genes involving STAT3 signaling pathway in parental versus their resistant cells by custom-designed high-throughput RCR array (Supplementary Tab. 1). The significant elevated expression of FN and its ligand β1 integrin was consistently observed in the two resistant cells compared to parental cells with a prominent upregulation of EGF and IL-6 in the respective BT474R and NCI-N87R cells (Supplementary Fig. 2A), which was further confirmed by real-time RT-PCR (Fig. 4A), conventional RT-PCR (Supplementary Fig. 2B) and western blotting (Supplementary Fig. 2C). The results from conventional RT-PCR (Supplementary Fig. 2B) and western blotting (Supplementary Fig. 2C) also confirmed the unchanged IL-10 and downregulated LIF expression as seen in RCR array (Supplementary Tab. 1), further validating the effectiveness of PCR array assay.

To validate the role of FN, EGF and IL-6 in causing STAT3 hyperactivation and consequent trastuzumab resistance, we stimulated parental cells with recombinant proteins or knocked down the expression of these molecules in resistant cells to evaluate STAT3 activation and trastuzumab sensitivity. Either FN, EGF or IL-6 stimulation alone enhanced STAT3 phosphorylation in parental BT474 and NCI-N87 cells with combined stimulations (FN/EGF or FN/IL-6) further increasing STAT3 activation, causing the resistance of these parental cells to trastuzumab treatment (Fig. 4B and 4C, left panel). Conversely, knockdown of FN EGF or IL-6 expression suppressed STAT3 phosphorylation in resistant BT474R and NCI-N87R cells, resulting in partial rescue of sensitivity of these cells to trastuzumab treatment (Fig. 4B and 4C, right panel). Combined silencing (FN/EGF or FN/IL-6) further inhibited the STAT3 activation and almost completely restored the trastuzumab sensitivity in resistant cells. However, no significant change in the trastuzumab sensitivity was seen when IL-10 expression was silenced in resistant cells (Supplementary Fig. 3), pointing to the importance of FN, EGF and IL-6 in STAT3-mediated trastuzumab resistance.

Notably, we found that silencing STAT3 expression also decreased the expression of FN/EGF and FN/IL-6 expression in the respective BT474R and NCI-N87R cells (Fig. 4D), indicating that a positive feedback loop between STAT3 and FN/EGF or FN/IL-6 is operated in these resistant cells which amplifies and fosters the STAT3 signal and consequently mediates trastuzumab resistance. Taken together, the data suggest that heterogeneous mediators converge on STAT3 hyperactivation contributing to trastuzumab resistance in our model.

### STAT3 upregulates the expression of MUC1 and MUC4 that mediates trastuzumab resistance

**Figure 5:**
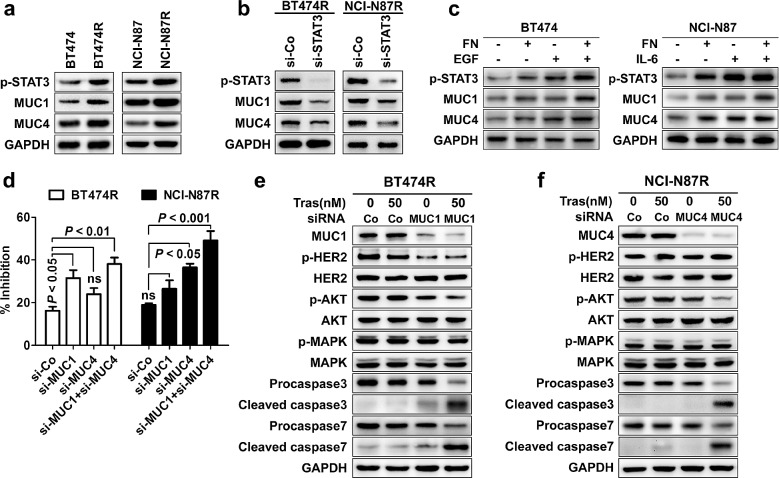
STAT3 upregulates the expression of MUC1 and MUC4 that mediates trastuzumab resistance (A) Immunoblots comparing the expression of MUC1 and MUC4 in trastuzumab-sensitive and -resistant cells. (B) Immunoblots detecting the expression of p-STAT3, MUC1 and MUC4 in BT474R and NCI-N87R cells after transfection with control or STAT3-specific siRNA. (C) Immunoblots assessing levels of STAT3 phosphorylation, MUC1 and MUC4 in parental BT474 cells upon FN and/or EGF stimulation (left) or in parental NCI-N87 cells upon FN and/or IL-6 treatment (right). (D) MTS assay evaluating cell proliferation of BT474R and NCI-N87R cells upon trastuzumab treatment after transfection with control (si-Co) or MUC1 (si-MUC1) and/or MUC4 (si-MUC4) specific siRNA. (E-F) Immunoblots assessing HER2 phosphorylation, major signaling molecules and apoptosis upon treatment with trastuzumab in BT474R cells with/without MUC1 knockdown (E) or in NCI-N87R cells with/without MUC4 knockdown (F). Data are expressed as mean ± SD of three independent experiments performed in triplicate samples, and picture is representative of three independent experiments.

To figure out the executioners of trastuzumab resistance induced by STAT3 hyperactivation, we analyzed the expression alteration of target genes downstream of STAT3 activation in trastuzumab-resistant cells relative to parental cells. From the above PCR array (Supplementary Fig. 2A), we found that MUC1 and MUC4, two STAT3 target genes previously implicated as a mediator of trastuzumab resistance in breast cancer [26-31], were most prominently upregulated in trastuzumab-resistant cells with a more preferential upregulation of MUC1 (2.83 fold) and MUC4 (8.49 fold) seen in BT474R and NCI-N87R cells respectively. Western blotting and immunohistochemistry confirmed the upregulation of MUC1 and MUC4 in resistant cells and their corresponding tumor xenografts at the protein levels respectively (Fig. 5A and Supplementary Fig. 1). Furthermore, silencing STAT3 expression decreased the expression of MUC1 and MUC4 in both BT474R and NCI-N87R cells (Fig. 5B); conversely, both FN/EGF and FN/IL-6 stimulations enhanced the STAT3 phosphorylation and consequently increased the expression of MUC1 and MUC4 in parental BT474 and NCI-N87 cells respectively (Fig. 5C). The data reveal that the expression of MUC1 and MUC4 is subjected to STAT3 signal in these cells.

To further define the role of MUC1 and MUC4 in mediating trastuzumab resistance, we attenuated their expression in resistant cells to determine whether trastuzumab sensitivity could be rescued. As shown in Fig. 5D, knockdown of MUC1 and MUC4 partially recovered the trastuzumab sensitivity in both BT474R and NCI-N87R cells with a more pronounced effect for MUC1 and MUC4 seen in the BT474R and NCI-N87R cells respectively, corresponding to their preferential upregulation in these two cell lines. Only upon MUC1 or MUC4 silencing, trastuzumab treatment suppressed the AKT phosphorylation and induced a prominent apoptosis in respective BT474R or NCI-N87R cells as evidenced by the appearance of cleaved caspase 3 and 7 and decreased levels of their precursor proteins (Fig. 5E and 5F), indicating MUC1 or MUC4 knockdown recovers the sensitivity of BT474R or NCI-N87R cells to trastuzumab treatment by decreasing the prosurvival AKT signal and increasing the apoptosis induction.

Previous studies have demonstrated that MUC1 and MUC4 mediated trastuzumab resistance predominantly by maintenance of persistent HER2 activation and masking of trastuzumab binding to HER2 respectively [26, 27, 31]. Consistent with previous findings, we found that MUC1 but MUC4 silencing inhibited the HER2 phosphorylation in BT474R cells (Fig. 5E and 5F), making them more susceptible to trastuzumab-induced apoptosis; however, knockdown of MUC4 expression in NCI-N87R cells significantly increased the binding of trastuzumab to HER2 (Supplementary Fig. 4). Collectively, STAT3 mediate the trastuzumab resistance via regulation of MUC1 and MUC4 expression in our model.

### STAT3 inhibition overcomes trastuzumab resistance *in vitro* and *in vivo*

**Figure 6:**
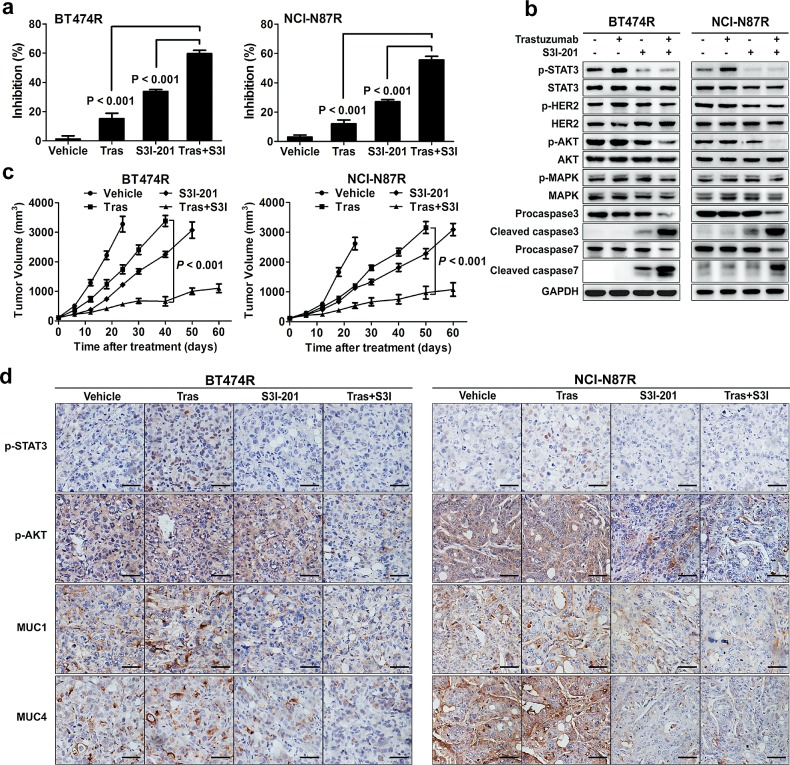
STAT3 inhibition overcomes trastuzumab resistance *in vitro* and *in vivo* (A) MTS assay evaluating the effects of trastuzumab (50 nM), STAT3 inhibitor (50 μM S3I-201) or combined treatment in BT474R and NCI-N87R cells. (B) Immunoblots evaluating HER2 phosphorylation, major signaling molecules and apoptosis in response to trastuzumab (50 nM), STAT3 inhibitor (50 μM S3I-201) or combined treatment in BT474R and NCI-N87R cells. (C) Tumor growth curves of BT474R and NCI-N87R xenografts upon treatment of either trastuzumab or STAT3 inhibitor S3I-201 alone or combination. (D) Representative histological staining of STAT3 and AKT phosphorylation as well as MUC1 and MUC4 expression after single or combined treatments in BT474R and NCI-N87R xenograft tumors. Scale bar, 100 μm. Data are expressed as mean ± SD of two independent experiments performed in triplicate samples, and pictures are representative of two independent experiments.

Since the data underscore a central role of STAT3 hyperactivation in mediating trastuzumab resistance, we hypothesized that targeting this pathway could be an effective strategy to overcome trastuzumab resistance. To target STAT3, we used S3I-201, an available small-molecule STAT3 inhibitor in preclinical studies [32]. As expected, S3I-201 effectively inhibited STAT3 activity and sensitized resistant BT474R and NCI-N87R cells to trastuzumab treatment *in vitro* (Fig. 6A). Accordingly, combined treatment of S3I-201 and trastuzumab suppressed AKT phosphorylation and caused a prominent apoptosis in resistant cells (Fig. 6B). Addition of S3I-201 also increased the suppressive efficacy of trastuzumab in parental BT474 and NCI-N87 cells (Supplementary Fig. 5).

To validate these results *in vivo*, mouse xenograft tumor models were established by subcutaneous injection of BT474R and NCI-N87R cells. As expected, trastuzumab treatment alone marginally suppressed the growth of BT474R and NCI-N87R xenografts *in vivo* (Fig. 6C); however, combined S3I-201 and trastuzumab treatment significantly retarded *in vivo* growth of BT474R and NCI-N87R xenografts. S3I-201 treatment alone also had a modest growth-suppressing effect in these two models. Immunohistochemistry of excised xenografts demonstrated that only combined treatment concomitantly inhibited the STAT3 phosphorylation and prosurvival AKT signal (Fig. 6D, upper two panels). Consistent with their modulation by STAT3 signal, MUC1 and MUC4 expression in excised xenografts were suppressed by either S3I-201 alone or combination treatment (Fig. 6D, bottom two panels). Notably, we did not observed severe side effects in combination treated mice as evidenced by their undisturbed body weight increase during the course of treatment (Supplementary Fig. 6) and lack of toxic manifestation in histological staining of their major organs at the terminus of experiments (Supplementary Fig. 7). Taken together, the data demonstrate that inhibition of STAT3 activity resensitizes trastuzumab-resistant to trastuzumab treatment *in vitro* and *in vivo* at least partially due to increased apoptosis induction, and combined treatment of trastuzumab and STAT3 inhibitor is an effective strategy to overcome trastuzumab resistance in this scenario.

## DISCUSSION

Although trastuzumab has resulted in a clinically and statistically significant benefit in patients with HER2-overexpressing breast and gastric cancer, many patients develop resistance after treatment, which ultimately culminates in treatment failure [33]. Despite multiple resistance mechanisms have been proposed in breast cancer, it remains unclear whether the similar mechanisms of trastuzumab resistance operate in gastric cancer. The investigation of trastuzumab resistance mechanisms and consequent design of relevantly therapeutic strategies is persistently under intensive research.

STAT3 appears to be a point of convergence for numerous oncogenic signaling pathways. Constitutive activation of STAT3 has been detected at high frequency in diverse human cancer cell lines and tissues, including breast cancer and gastric cancer [34-36]. Suppression of STAT3 activity by small-molecule inhibitors leads to increased apoptosis, chemosensitivity, and decreased angiogenesis in cancer cells with constitutively activated STAT3 [32, 37]. Here, we provide strong evidence that increased STAT3 activity confers *de novo* and acquired resistance to trastuzumab in our model system. This mechanism of trastuzumab resistance has not been previously identified in HER2-positive gastric cancer model, although recent studies have shown that STAT3 activation promotes epithelial-mesenchymal transition and expansion of cancer stem cells (CSCs) in breast cancer [22-24], which may contribute to trastuzumab resistance. We further demonstrate that inhibition of STAT3 by siRNA-mediated knockdown or a small molecule inhibitor S3I-201 universally sensitized trastuzumab-resistant cells to trastuzumab treatment and significantly suppressed tumor growth *in vitro* and *in vivo*. The superior antiproliferative and antitumor effect of STAT3 inhibition in combination with trastuzumab concurred with its ability to promote cell apoptosis and reduce AKT phosphorylation both *in vitro* and *in vivo*. In addition, we noted that even in the trastuzumab-sensitive parental cells, addition of STAT3 inhibitor resulted in a greater magnitude of response compared with trastuzumab alone. This may not be surprising, given the universe presence of constitutive activation of STAT3 signaling in these cancer cells and their well-characterized prosurvival effect. This novel combination strategy warrants further clinical investigation in patients with trastuzumab-resistant tumors given that constitutively activated STAT3 is present in a quite fraction of patients with HER2-positive breast and gastric cancer [38, 39].

**Figure 7:**
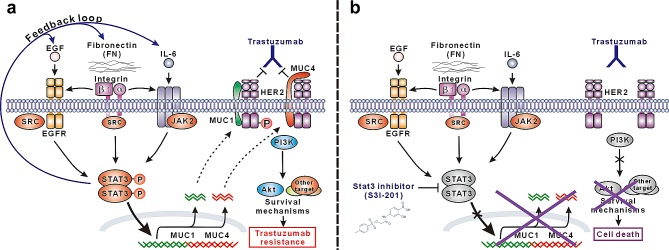
Schematic presentation of the molecular mechanism of trastuzumab resistance mediated by STAT3-dependent feedback loop and corresponding targeting strategy (A) STAT3 is activated through multiple signaling pathways in our model, including SRC-dependent EGF:EGFR:STAT3 signaling, FN:EGFR:STAT3 signaling, and IL-6:JAK2:STAT3 signaling. Activated STAT3 promotes the expression of FN, EGF and IL-6 and therefore constitutes a positive feedback loop to persistently maintain STAT3 signaling, which leads to the upregulation of MUC1 and MUC4 mediating trastuzumab resistance via sustaining HER2 phosphorylation and/or blocking trastuzumab binding. (B) In STAT3 hyperactivation-mediated trastuzumab-resistant cancer cells, STAT3-specific small-molecule inhibitor S3I-201 efficiently inhibits STAT3 activation and consequent expression of MUC1 and MUC4, which recovers trastuzumab sensitivity leading to suppression of HER2 signaling, downstream AKT activity and finally apoptosis in response to trastuzumab treatment.

Many factors in tumors have been shown to transiently or persistently activate STAT3 signaling that is known to regulate the expression of specific target genes and has important roles in tumorigenesis. To determine the key upstream factors and downstream target genes responsible for STAT3-mediated trastuzumab resistance in current models, we compared the expression of 91 known genes involving STAT3 signaling pathway in both resistant cells over their parental cells by high-throughput PCR array. Besides selective elevation of EGF and IL-6, two well-known STAT3 trigger genes, in respective BT474R and NCI-87R cells, FN and its ligand β1 integrin were shown to be consistently upregulated in both resistant cell lines, whose expression were further confirmed at mRNA and protein levels by RT-PCR and western blotting. Recent studies have shown that β1 integrin overexpression or engagement by FN activates STAT3 signaling pathway via JAK2, SRC or FAK adaptor proteins, which participates in breast cancer progression or their resistance to HER2-targeting small-molecule inhibitors [40, 41], as has found β1 integrin in mediating resistance of lung cancer to EGFR-targeting drugs [42, 43]. FN-β1 integrin signal was also found to synergistically enhance STAT3 activation induced by EGF and IL-6 in breast or other cancer cells [40, 44]. In addition, we observed that silencing STAT3 decreased the expression of FN, EGF and IL-6 in resistant cells; thus, it is reasonable to speculate that a positive feedback loop involving FN/EGF/STAT3 or FN/IL-6/STAT3 is operative in BT474R or NCI-87R resistant cells respectively, which leads to the propagation and amplification of STAT3 signaling contributing to trastuzumab resistance (Fig. 7A).

Our data show that the expression of MUC1 and MUC4, two STAT3 target genes [45, 46], was markedly increased in resistant cells compared to their parental cells, and was upregulated in parental cells in response to stimulations from FN, EGF and IL-6 via STAT3 pathway. MUC1 and MUC4 have been previously described to mediate trastuzumab resistance via promotion of HER2 activation and blockade of trastuzumab binding respectively in breast and gastric cancer [27, 31]. Consistent with previous findings, MUC1 knockdown inhibited HER2 activation in resistant BT474R cells, resensitizing them to trastuzumab-induced growth suppression and apoptosis. Likewise, MUC4 silencing derepressed the hindrance of trastuzumab binding to HER2 leading to the partial recovery of trastuzumab sensitivity in resistant NCI-87R cells. Silencing both MUC1 and MUC4 further produced a synergistic effect, resulting in an almost recovery of trastuzumab sensitivity in both resistant cells. These results indicate that self-maintained persistent STAT3 signal mediates trastuzumab resistance via upregulating the expression of MUC1 and MUC4 in our model (Fig. 7A).

Emerging evidence suggests that STAT3 activation can induce epithelial-mesenchymal transition and consequently propagate cancer stem cells (CSCs) that have been described to be responsible for HER2-targeting drug resistance and ultimate tumor recurrence in breast and gastric cancers [22-25]. Thus, it will be interesting to learn whether a similar mechanism is operative in our model in future studies.

In conclusion, our work proposes a possible mechanism of trastuzumab resistance fostered by a STAT3-centered positive feedback loop comprising of FN/EGF/IL-6 upstream mediators and MUC1/4 downstream executioners in HER2-positive breast and gastric cancer, providing a rationale for applying STAT3 inhibitors to overcome trastuzumab resistance (Fig. 7B). We acknowledge that studies need to be done to further define the role of intratumoral STAT3 activation in mediating trastuzumab resistance using clinical tumor samples. It may be a good start to retrospectively explore the correlation between STAT3 phosphorylation and therapy response in patients with HER2-overexpressing breast and gastric cancer.

## MATERIALS AND METHODS

### Cell culture, plasmids and small interfering RNA (siRNA)

BT474 breast cancer cell line and NCI-N87 gastric cancer cell line were obtained from the American Type Culture Collection (ATCC, Manassas, VA, USA). BT474R and NCI-N87R cells were selected for resistance to trastuzumab by continuously culturing parental cells with increasing doses of trastuzumab for longer than 15 months as previously described.[47] The plasmid pcDNA3.1-STAT3-CA (constitutively activated mutant of STAT3) was obtained from Addgene (Cambridge, MA, USA). Cells were transiently transfected with siRNA using Lipofectamine 2000 (Invitrogen, Carlsbad, CA) according to the manufacturer's protocol. The siRNA specific for STAT3, PTEN, MUC1 and control siRNA (si-Co) were obtained from Cell Signaling Technology (Denvers, MA, USA). FN, IL-6, IL-10, EGF and MUC1/4 targeting siRNA were purchased from Santa Cruz Biotechnology (Santa Cruz, CA, USA). For assessing the effect of FN stimulation, cells were incubated in serum-free medium onto culture plates pro-coated with 50 μg/ml of FN (Sigma-Aldrich, St Louis, MO, USA) or bovine serum albumin (BSA, Sigma-Aldrich) for 12 h. For EGF and IL-6 stimulations, cells were treated with 20 ng/ml EGF or IL-6 (R&D Systems, Minneapolis, MN, USA) for 2 h.

### Cell growth inhibition assay

A total of 3000 cells were seeded in triplicate into 96-well plates. After 12 h, the medium in the wells was replaced with fresh medium containing drugs as indicated. In some experiments, the cells were transfected with gene-specific siRNA, 24 h later, were subjected to serum starvation overnight, and seeded in serum-free medium onto FN-coated plates for 12 h, with culture onto BSA-coated plates as controls. After treatment for an additional 4 d, cell viability was determined using CellTiter 96 AQueous One Solution Cell Proliferation Assay kit (Promega, Madison, Wisconsin) and calculated with GraphPad Prism software (Castro Valley, CA, USA). Cell inhibition (%) was calculated as follows: (1 - experimental absorbance/control absorbance) × 100.

### Conventional and real-time quantitative reverse transcriptase (RT)-PCR

A total of 1 × 10^6^ cells were collected for each sample. The total RNA was isolated from cells using TRIzol reagent (Invitrogen). cDNAs were synthesized by Maxima First Strand cDNA Synthesis Kit for RT-qPCR (Thermo Scientific, Rockford, IL, USA) in accordance with the manufacturer's instructions. Conventional RT-PCR was used to detect the expression of FN and its integrin receptors. Amplification of GAPDH was used as the control. The differences in gene expression between parental and trastuzumab-resistant cell lines were validated by real-time quantitative RT-PCR (qPCR) using GoTaq qPCR Master Mix with SYBR green (Promega) on Applied Biosystems 7500 Real-Time PCR System (Applied Biosystems, Foster City, USA) as recommended by the manufacturer. The data were analyzed using the comparative cycle threshold (Ct) method with GAPDH as an internal control. The primer sequences are available upon request.

### PCR array

96-well custom PCR array (Qiagen, Shanghai, China) was designed to assess gene expression changes of resistant cells relative to parental cells, in which 91 genes involved in the activation and downstream effects of STAT3 signaling and five housekeeping genes were included (see Supplementary Table 1). The PCR array was performed and analyzed according to the instruction from manufacturer.

### *In vivo* tumor model

Female athymic nude mice (5-6 weeks old; Weitonglihua Biotechnology, Beijing, China) were maintained and treated under specific pathogen-free conditions. Mice were injected in the left flank area subcutaneously (s.c.) with 5 × 10^6^ indicated tumor cells in 100 μl of PBS. When xenografts reached a diameter of 4-5 mm, animals were randomized into different treatment groups (5 mice per group) with similar mean tumor sizes in each group. Mice were intraperitoneally (i.p.) given trastuzumab (10 mg/kg in 100 μl PBS) or vehicle control (PBS) once per week and/or intravenously (i.v.) administered with S3I-201 (5 mg/kg in DMSO) thrice weekly. Mice were monitored for tumor growth and tumor volume was calculated according to the formula: volume =π/6 × a^2^ × b, where a is the smallest superficial diameter and b is the largest superficial diameter.

### Western blotting analysis

The whole-cell protein lysates or animal tumor tissue specimens were prepared as previously described [47] and analyzed by western blot with the following primary antibodies: phosphorylated STAT3 (p-STAT3, Tyr705), phosphorylated AKT (p-AKT, Ser473), phosphorylated MAPK (p-MAPK, Thr202/Tyr204), phosphorylated HER2 (p-HER2, Tyr1221/1222), phosphorylated EGFR (p-EGFR, Tyr1068), phosphorylated HER3 (p-HER3, Tyr1289), phosphorylated IGF-IR (p-IGF-IR, Tyr1316), STAT3, AKT, MAPK, EGFR, HER2, HER3, IGF-IR, PTEN, caspase 3 and 7 from Cell Signaling Technology (Denvers, MA, USA); FN, IL-6, IL-10, LIF, EGF, MUC1 (clone EP1024Y) and MUC4 (clone 8G-7) from Abcam (Cambridge, MA, USA).

### Immunohistochemistry

Immunohistochemical staining of phosphorylated AKT, phosphorylated STAT3, MUC1 and MUC4 was done as previously described.[47] Briefly, tumors were harvested from mice 40 d after treatment and fixed in 10% formalin, from which 4 μm-thick sections were made. The slides were incubated with 3% hydrogen peroxide to quench endogenous peroxidase activities. After heat-induced antigen retrieval, the specimens were blocked with normal goat serum for 1 h at room temperature, and incubated with the antibodies against p-AKT (Ser473), p-STAT3 (Tyr705), MUC1 and MUC4 using a predefined optimal concentration at 4 °C overnight. Slides underwent color development with 3-3'-diaminobenzidine (DAB) and were counterstained with hematoxylin. Photomicrographs were taken with an Olympus microscope BX51.

### Flow cytometric analysis

1 × 10^6^ cells were collected gently and incubated with trastuzumab (10 μg/ml) or control IgG at 4 °C for 30 min. After washing three times with PBS, the cells were incubated with Alexa Fluor 488 goat anti-mouse IgG (Invitrogen) at 4 °C for 30 min. After washing, cells were resuspended in PBS containing 1% formaldehyde. The fluorescence intensity was detected by Cytomics FC 500 MPL flow cytometer (Beckman Coulter, Fullerton, CA, USA) and analyzed using FlowJo software (Tree Star, Inc., CA, USA).

### Statistical analysis

The experiments were repeated at least two times. All data were expressed as mean ± SD and examined by Student's t-test for statistical significance. p<0.05 was considered statistically significant.

## SUPPLEMENTARY INFORMATION FIGURES AND TABLE


