# Supplementary material for: Feedback activation of STAT3 mediates trastuzumab resistance via upregulation of MUC1 and MUC4 expression
Source: Oncotarget. 2014 Jun 26;5(18):8317–29. doi: 10.18632/oncotarget.2135 (PMC4226685; doi:10.18632/oncotarget.2135)
Supplement: Supplementary file 1 [file oncotarget-05-8317-s001.pdf]

Feedback activation of STAT3 mediates trastuzumab resistance via upregulation of MUC1 and MUC4 expression

Supplementary Information

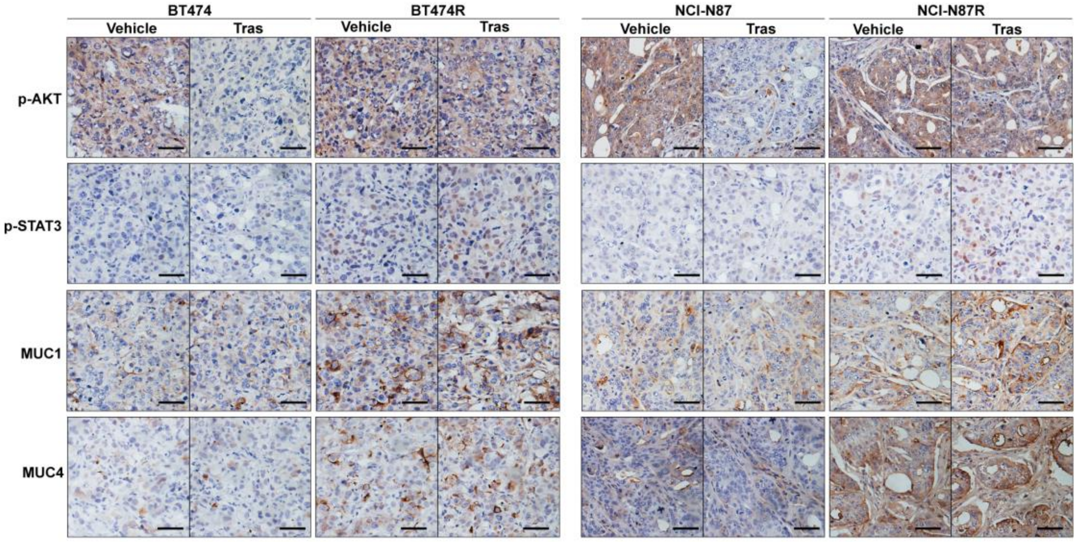

Supplementary Figure 1: Representative graphs showing immunohistological staining of phosphorylated AKT (Ser473), phosphorylated STAT3 (Tyr705), MUC1 and MUC4 in parental and resistant tumor xenografts from mice treated with control or trastuzumab. Scale bar, 100 μm. Pictures are representative of two independent experiments.

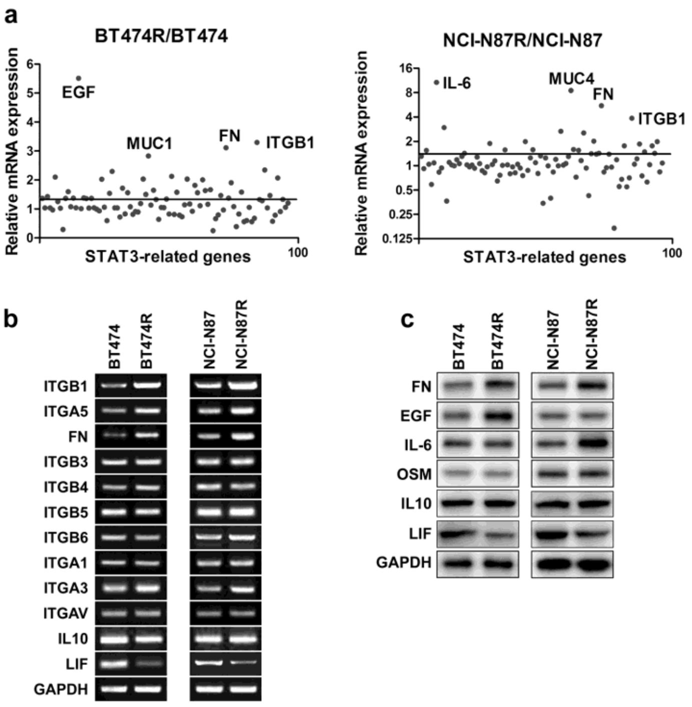

Supplementary Figure 2: Gene expression changes between parental and trastuzumab-resistant cell lines. (A) Scatter plot of fold-change of STAT3-related genes in trastuzumab-resistant versus parental cell lines with mean value (black line). (B) Comparison of gene expression of FN and its integrin receptors as well as STAT3-related IL-10 and LIF between parental and trastuzumab-resistant cell lines by conventional RT-PCR. (C) Immunoblots comparing the expression of FN, EGF and IL-6 as well as STAT3-related IL-10 and LIF in the two resistant cells and their parental cells. Data are representative of two independent experiments.

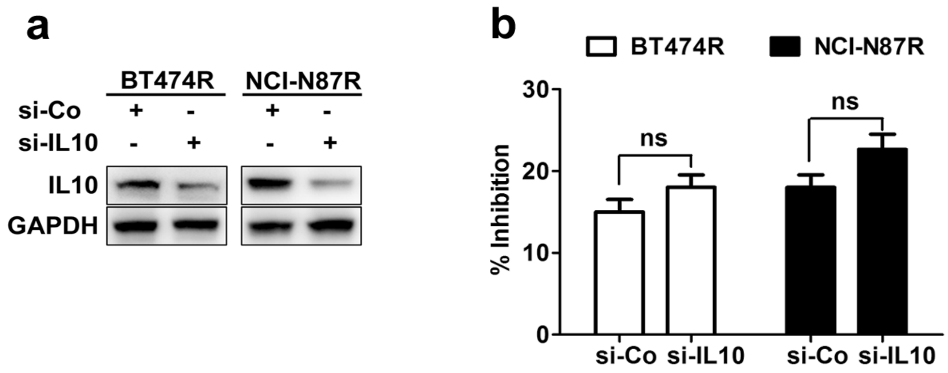

Supplementary Figure 3: Histograms showing the binding of trastuzumab to NCI-N87 (upper panels) and NCI-N87R (bottom panels) cells after MUC4 knockdown or STAT3 inhibition. NCI-N87 and NCI-N87R cells were transfected with the control (si-Co) or MUC4 siRNA (si-MUC4) or treated with 50 μM S3I-201. Twenty-four hours later, cells were collected and stained with isotype control antibody (ISO) or trastuzumab (10 μg/ml) at 4 ˚C for 30 min, followed by addition of Alexa Fluor 488-conjugated secondary antibody. After washing, the fluorescence intensity was detected by flow cytometry. Data are representative of two independent experiments.

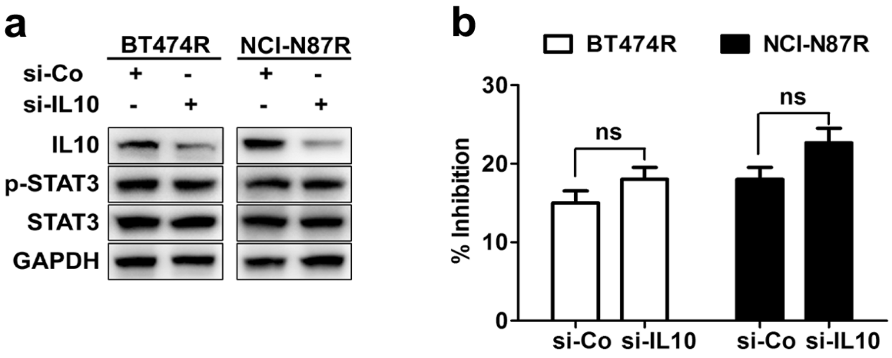

Supplementary Figure 4: Effects of IL-10 on the sensitivity of resistant cells to trastuzumab. IL-10 was silenced in BT474R and NCI-N87R cells **(a)**, and the cell growth inhibition by trastuzumab was determined by MTS assay **(b)**.

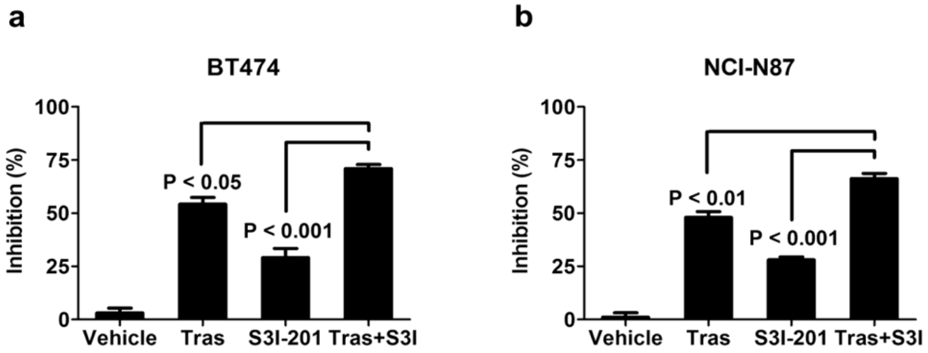

Supplementary Figure 5: MTS assay examining the effects of trastuzumab (50 nM), STAT3 inhibitor (50 μM S3I-201) or combined treatment in parental BT474 **(a)** and NCI-N87 **(b)** cell lines. Data are expressed as mean ± SD of two independent experiments performed in triplicate samples.

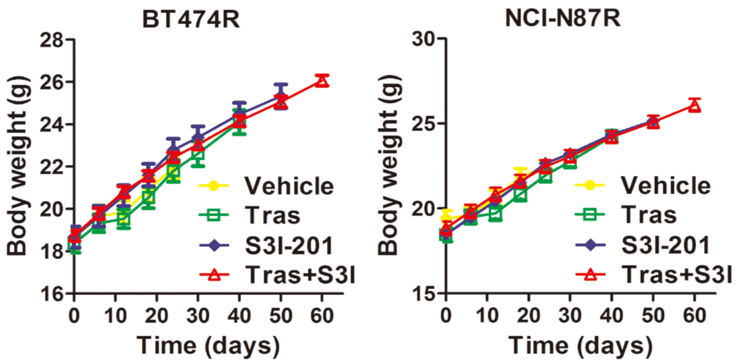

Supplementary Figure 6: The mice bearing subcutaneous BT474R and NCI-N87R xenografts were treated with vehicle control, trastuzumab, S3I-201 and trastuzumab/ S3I-201. Body weight of mice was measured during the course of treatment.

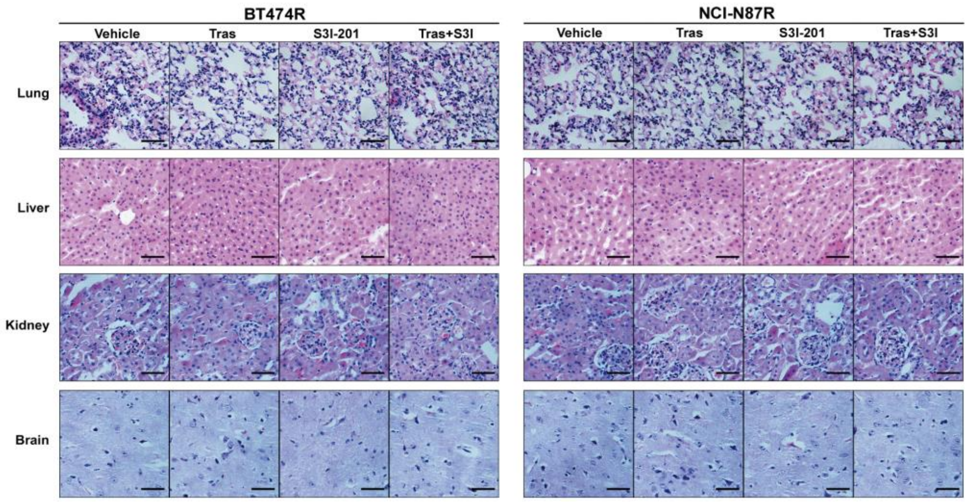

Supplementary Figure 7: The representative graphs of hematoxylin and eosin (H&E) staining of the lung, liver, kidney and brain tissues harvested from treated mice at the terminus of experiments.

Table 1: The relative fold expression of key genes involved in the activation and downstream effects of STAT3 signaling.

	GENES	BT474R:BT474	NCI-N87R:NCI-N87
1	**EGF**	**5.512**	1.186
2	**Fibronectin (FN)**	**3.111**	**5.512**
3	**ITGB1**	**3.297**	**3.868**
4	**MUC1**	**2.834**	**1.983**
5	**MUC4**	**1.819**	**8.487**
6	**IL6**	1.257	**10.684**
7	18S rRNA	1.039	0.998
8	AKT1	1.369	1.309
9	Bad	1.426	1.544
10	Bax	0.968	0.5874
11	Bcl2	1.03	0.831
12	beta-actin	1.036	1.042
13	beta-tubulin	1.099	0.995
14	CCL2 (MCP-1)	1.366	2.954
15	CCL3 (MIP-1A)	0.294	0.938
16	CCL4 (MIP-1B)	1.38	1.021
17	CCL5 (RANTES)	1.36	1.423
18	CCND1	1.076	1.098
19	CD40 (TNFRSF5)	1.063	0.869
20	CD40LG (TNFSF5)	0.597	0.817
21	CDKN1A (p21)	1.541	0.968
22	CDKN1B (p27)	2.295	1.878
23	c-fos	2.104	1.084
24	CRP	1.0341	1.025
25	CSF1 (MCSF)	1.365	0.965
26	CSF2 (GM-CSF)	1.045	1.017
27	CSF3 (GCSF)	0.993	0.93
28	CSF3R (GM-CSFR)	0.938	1.276
29	CXCL10 (INP10)	2.028	1.058
30	Cyclin D1	1.039	1.147
31	Cyclin E	1.338	1.034

32	Cyclophilin	1.069	1.002
33	FAS (TNFRSF6)	1.012	1.06
34	FASLG (TNFSF6)	1.384	1.304
35	GAPDH	1.041	0.998
36	HGF	0.922	1.346
37	IL10	0.83	1.244
38	IL11	2.128	0.996
39	IL13	0.814	0.397
40	IL15	1.575	0.835
41	IL17A	0.785	1.194
42	IL18	0.731	0.995
43	IL18R1	1.143	2.662
44	IL1A	1.716	0.764
45	IL1B	0.835	1.007
46	IL1R1	1.562	1.567
47	IL2	1.076	1.024
48	IL21	2.032	1.018
49	IL22	1.175	1.063
50	IL23A	0.921	1.294
51	IL2RA (CD25)	0.819	0.795
52	IL3	2.032	0.935
53	IL4	0.899	0.817
54	IL5	1.187	1.044
55	IL6R	1.055	0.802
56	IL6ST (GP130)	1.048	1.321
57	IL8	1.34	0.343
58	ITGA1	1.137	0.875
59	ITGA3	1.399	1.207
60	ITGA5	1.869	2.189
61	ITGAV	0.588	0.928
62	ITGB3	1.328	1.042
63	ITGB4	1.289	1.215
64	ITGB5	2.35	1.85
65	ITGB6	1.038	0.749
66	JAK2	1.563	1.585
67	JAK3	1.111	0.721
68	JUNB	2.005	1.547
69	LIF	0.255	0.427
70	LIFR	0.667	1.407
71	LTA (TNFB)	1.163	2.534
72	MAP2K1 (MEK1)	0.826	0.169
73	MAPK1 (ERK2)	1.581	1.185
74	MAPK14	0.561	0.552
75	MAPK3 (ERK1)	0.604	0.679
76	MAPK8 (JNK1)	1.059	1.758

77	MET	0.968	1.421
78	MTOR	1.505	0.755
79	MUC2	1.638	1.16
80	MUC3	1.667	0.934
81	MUC5	0.792	0.963
82	MYC	1.375	0.833
83	OSM	1.084	1.386
84	PIAS3	0.565	0.549
85	PIM1	0.68	0.706
86	SOCS1	1.646	1.252
87	SOCS3	0.627	0.573
88	SRC	0.645	1.09
89	STAT3	0.971	1.116
90	Survivin	1.306	1.146
91	TNF	0.785	1.029
92	TNFRSF10B (DR5)	2.073	1.759
93	TNFRSF1A	0.913	1.42
94	TNFRSF1B	1.334	1.984
95	TNFSF10 (TRAIL)	1.056	0.837
96	TYK2	1.203	1.076

NOTE: This array includes activators, downstream mediators, and target genes for STAT3 signaling. The expression of a focused panel of genes was detected by real-time PCR.
